# Ion-induced nucleation of pure biogenic particles

**DOI:** 10.1038/nature17953

**Published:** 2016-05-25

**Authors:** Jasper Kirkby, Jonathan Duplissy, Kamalika Sengupta, Carla Frege, Hamish Gordon, Christina Williamson, Martin Heinritzi, Mario Simon, Chao Yan, João Almeida, Jasmin Tröstl, Tuomo Nieminen, Ismael K. Ortega, Robert Wagner, Alexey Adamov, Antonio Amorim, Anne-Kathrin Bernhammer, Federico Bianchi, Martin Breitenlechner, Sophia Brilke, Xuemeng Chen, Jill Craven, Antonio Dias, Sebastian Ehrhart, Richard C. Flagan, Alessandro Franchin, Claudia Fuchs, Roberto Guida, Jani Hakala, Christopher R. Hoyle, Tuija Jokinen, Heikki Junninen, Juha Kangasluoma, Jaeseok Kim, Manuel Krapf, Andreas Kürten, Ari Laaksonen, Katrianne Lehtipalo, Vladimir Makhmutov, Serge Mathot, Ugo Molteni, Antti Onnela, Otso Peräkylä, Felix Piel, Tuukka Petäjä, Arnaud P. Praplan, Kirsty Pringle, Alexandru Rap, Nigel A. D. Richards, Ilona Riipinen, Matti P. Rissanen, Linda Rondo, Nina Sarnela, Siegfried Schobesberger, Catherine E. Scott, John H. Seinfeld, Mikko Sipilä, Gerhard Steiner, Yuri Stozhkov, Frank Stratmann, Antonio Tomé, Annele Virtanen, Alexander L. Vogel, Andrea C. Wagner, Paul E. Wagner, Ernest Weingartner, Daniela Wimmer, Paul M. Winkler, Penglin Ye, Xuan Zhang, Armin Hansel, Josef Dommen, Neil M. Donahue, Douglas R. Worsnop, Urs Baltensperger, Markku Kulmala, Kenneth S. Carslaw, Joachim Curtius

**Affiliations:** 1 Goethe University Frankfurt, Institute for Atmospheric and Environmental Sciences, Frankfurt am Main, 60438 Germany; 2 CERN, Geneva, CH-1211 Switzerland; 3 Department of Physics, University of Helsinki, Helsinki, FI-00014 Finland; 4 Helsinki Institute of Physics, University of Helsinki, Helsinki, FI-00014 Finland; 5 School of Earth and Environment, University of Leeds, Leeds, LS2 9JT UK; 6 Paul Scherrer Institute, Laboratory of Atmospheric Chemistry, Villigen, CH-5232 Switzerland; 7 Institute for Ion and Applied Physics, University of Innsbruck, Innsbruck, 6020 Austria; 8 Onera—The French Aerospace Lab, Palaiseau, F-91123 France; 9 SIM, University of Lisbon, Lisbon, 1849-016 Portugal; 10 Ionicon Analytik GmbH, Innsbruck, 6020 Austria; 11 Institute for Atmospheric and Climate Science, ETH Zurich, CH-8092 Zurich Switzerland; 12 Division of Chemistry and Chemical Engineering, California Institute of Technology, Pasadena, 91125 California USA; 13 WSL Institute for Snow and Avalanche Research SLF, Davos, CH-7260 Switzerland; 14 University of Eastern Finland, Kuopio, FI-70211 Finland; 15 Finnish Meteorological Institute, Helsinki, FI-00101 Finland; 16 Solar and Cosmic Ray Research Laboratory, Lebedev Physical Institute, Moscow, 119991 Russia; 17 University of Leeds, National Centre for Earth Observation, Leeds, LS2 9JT UK; 18 Department of Applied Environmental Science, University of Stockholm, Stockholm, SE-10961 Sweden; 19 Faculty of Physics, University of Vienna, Vienna, 1090 Austria; 20 Leibniz Institute for Tropospheric Research, Leipzig, 04318 Germany; 21 University of Beira Interior, Covilhã, 6201-001 Portugal; 22 Center for Atmospheric Particle Studies, Carnegie Mellon University, Pittsburgh, 15213 Pennsylvania USA; 23 Aerodyne Research Inc., Billerica, 01821 Massachusetts USA; 24 Present Address: † Present addresses: CIRES, University of Colorado Boulder, Boulder, Colorado 80309, USA (C.W.); Arctic Research Center, Korea Polar Research Institute, Incheon 406-840, South Korea (J. Kim); Department of Atmospheric Sciences, University of Washington, Seattle, Washington 98195, USA (S.S.)., ,

**Keywords:** Atmospheric chemistry, Climate-change impacts

## Abstract

Atmospheric aerosols and their effect on clouds are thought to be important for anthropogenic radiative forcing of the climate, yet remain poorly understood^1^. Globally, around half of cloud condensation nuclei originate from nucleation of atmospheric vapours^2^. It is thought that sulfuric acid is essential to initiate most particle formation in the atmosphere^3,4^, and that ions have a relatively minor role^5^. Some laboratory studies, however, have reported organic particle formation without the intentional addition of sulfuric acid, although contamination could not be excluded^6,7^. Here we present evidence for the formation of aerosol particles from highly oxidized biogenic vapours in the absence of sulfuric acid in a large chamber under atmospheric conditions. The highly oxygenated molecules (HOMs) are produced by ozonolysis of α-pinene. We find that ions from Galactic cosmic rays increase the nucleation rate by one to two orders of magnitude compared with neutral nucleation. Our experimental findings are supported by quantum chemical calculations of the cluster binding energies of representative HOMs. Ion-induced nucleation of pure organic particles constitutes a potentially widespread source of aerosol particles in terrestrial environments with low sulfuric acid pollution.

**Supplementary information:**

The online version of this article (doi:10.1038/nature17953) contains supplementary material, which is available to authorized users.

## Abstract (web summary)

Aerosol particles can form in the atmosphere by nucleation of highly oxidized biogenic vapours in the absence of sulfuric acid, with ions from Galactic cosmic rays increasing the nucleation rate by one to two orders of magnitude compared with neutral nucleation.

**Supplementary information:**

The online version of this article (doi:10.1038/nature17953) contains supplementary material, which is available to authorized users.

## Aerosol particle formation in clean air

The effect of atmospheric aerosols on clouds and the radiative forcing of the climate system remains poorly understood. It is thought that nucleation of aerosol particles from atmospheric vapours rarely proceeds in the absence of sulfuric acid. Now two papers in this week’s *Nature* point to a previously unappreciated role for highly oxygenated molecules (HOMs) in promoting new particle formation and growth, essentially a mechanism that produces aerosols in the absence of pollution. Jasper Kirkby *et al*. show that aerosol particles can form as a result of ion-induced nucleation of HOMs in the absence of sulfuric acid under conditions relevant to the atmosphere in the CLOUD chamber at CERN. Jasmin Tröstl *et al*. examined the role of organic vapours in the initial growth of nucleated organic particles in the absence of sulfuric acid in the CERN CLOUD chamber under atmospheric conditions. They find that the organic vapours driving initial growth have extremely low volatilities. With increasing particle size, subsequent growth is primarily due to more abundant organic vapours of slightly higher volatility.

**Supplementary information:**

The online version of this article (doi:10.1038/nature17953) contains supplementary material, which is available to authorized users.

## Main

It is thought that aerosol particles rarely form in the atmosphere without sulfuric acid^3,4^, except in certain coastal regions where iodine oxides are involved^8^. Furthermore, ions are thought to be relatively unimportant in the continental boundary layer, accounting for only around 10% of particle formation^5^. Sulfuric acid derives from anthropogenic and volcanic sulfur dioxide emissions as well as dimethyl sulfide from marine biota. However, typical daytime sulfuric acid concentrations (10^5^–10^7^ cm^−3^, or 0.004–0.4 parts per trillion by volume (p.p.t.v.) at standard conditions) are too low for sulfuric acid and water alone to account for the particle formation rates observed in the lower atmosphere^9^, so additional vapours are required to stabilize any embryonic sulfuric acid clusters against evaporation. Base species such as amines can do this and can explain part of atmospheric particle nucleation^10^. It is well established that oxidation products of volatile organic compounds (VOCs) are important for particle growth^11^, but whether their role in the smallest particles is in nucleation or growth alone has remained ambiguous^4,12,13^. Recently, however, it has been shown that oxidized organic compounds do indeed help to stabilize sulfuric acid clusters and probably play a major role in atmospheric particle nucleation^6,14,15^. We refer to these compounds as HOMs (highly oxygenated molecules) rather than ELVOCs (extremely low-volatility organic compounds)^16^ because the measured compounds span a wide range of low volatilities.

Here we report atmospheric particle formation solely from biogenic vapours. The data were obtained at the CERN CLOUD chamber (Cosmics Leaving OUtdoor Droplets; see Methods for experimental details) between October 2012 and November 2013. In contrast with other works that have reported organic particle formation without intentional addition of sulfuric acid^6,7^, here we measure the cluster chemistry and the role of ions, and rule out contamination.

Precursor VOCs in the atmosphere arise predominantly from natural sources such as vegetation and largely comprise isoprene (C_5_H_8_), monoterpenes (C_10_H_16_), sesquiterpenes (C_15_H_24_) and diterpenes (C_20_H_32_). Here we have studied α-pinene (C_10_H_16_) because it is the most abundant monoterpene, often exceeding 50 p.p.t.v. in the continental boundary layer^17^. We oxidized α-pinene by exposure to ozone and also to hydroxyl radicals (OH·) produced from ozone photolysis and secondary reactions. To measure the relative importance of these oxidants we also performed a few pure ozonolysis experiments (in which we removed OH· with a 0.1% H_2_ scavenger) and a few pure hydroxyl experiments (in which we generated OH· by photolysis of gas-phase nitrous acid, HONO). Two nitrate chemical ionization atmospheric pressure interface time-of-flight (CI-APi-TOF) mass spectrometers measured neutral gas-phase compounds in the chamber (H_2_SO_4_ and HOMs). Therefore, for this study, HOMs are implicitly defined as oxidized organic compounds that can be detected by a nitrate CI-APi-TOF; related molecules with a lower oxidation state or different functional groups could be present in the chamber, but undetected by our nitrate chemical ionization set-up.

**Extended Data Figure 1:**
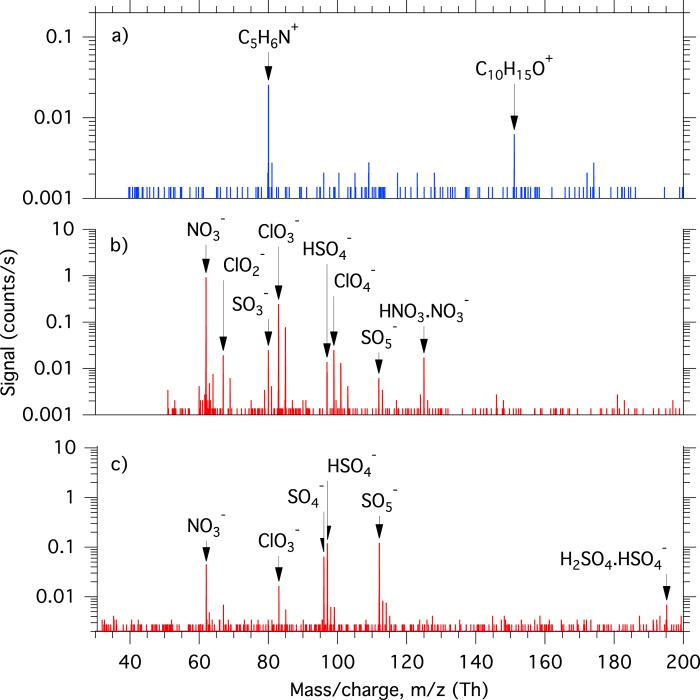
Small-ion mass spectra. **a**, **b**, Composition of positive (**a**) and negative (**b**) small ions measured by the APi-TOF under GCR conditions and before adding any SO_2_ to the chamber. The experimental conditions are zero α-pinene, 35 p.p.b.v. O_3_, zero H_2_ or HONO, 38% relative humidity, 278 K and [H_2_SO_4_] < 5 × 10^4^ cm^−3^. Collisions will transfer positive charge to contaminant molecules having the highest proton affinity (**a**), and negative charge to contaminant molecules with the lowest proton affinity, that is, highest gas-phase acidity (**b**). From molecular cluster measurements, the positive ions also include ammonium (), but its mass is below the set acceptance cut-off of the APi-TOF. **c**, The negative small-ion spectrum at [H_2_SO_4_] = 1.2 × 10^5^ cm^−3^, after adding 32 p.p.t.v. SO_2_ to the chamber, showing that the dominant ions species shift from nitrate to sulfur-containing. The experimental conditions are 340 p.p.t.v. α-pinene, 35 p.p.b.v. O_3_, zero H_2_ or HONO, 38% relative humidity and 278 K. Water molecules evaporate rapidly from most hydrated ions in the APi-TOF and so are not detected.

Before starting measurements, we carefully cleaned the CLOUD chamber (see Methods) and established extremely low contaminant concentrations: at 38% relative humidity and 278 K, the contaminants were below the detection limit for SO_2_ (<15 p.p.t.v.) and H_2_SO_4_ (<5 × 10^4^ cm^−3^), and total organics (largely comprising high volatility C_1_–C_3_ compounds) were below 150 p.p.t.v. Contaminants with a high proton affinity or a high gas-phase acidity can be detected as ions by the APi-TOF operating in positive or negative mode, respectively, even at neutral molecule concentrations as low as 10^4^ cm^−3^. The APi-TOF measured contaminant C_5_H_5_NH^+^ (protonated pyridine) and contaminant  to be the dominant positive and negative ions, respectively, before we added any trace gases to the chamber other than water vapour and ozone (Extended Data Fig. 1a, b). Despite its higher gas-phase acidity, we detected contaminant  at only 1% of the  signal (Extended Data Fig. 1b), ruling out any contribution of sulfuric acid to the nucleation measurements. From previous studies and molecular analysis of the charged clusters (see below), the most abundant positive ion is likely to be contaminant ammonium (), but its mass is below the acceptance cut-off of the APi-TOF as operated in this study.

**Figure 1:**
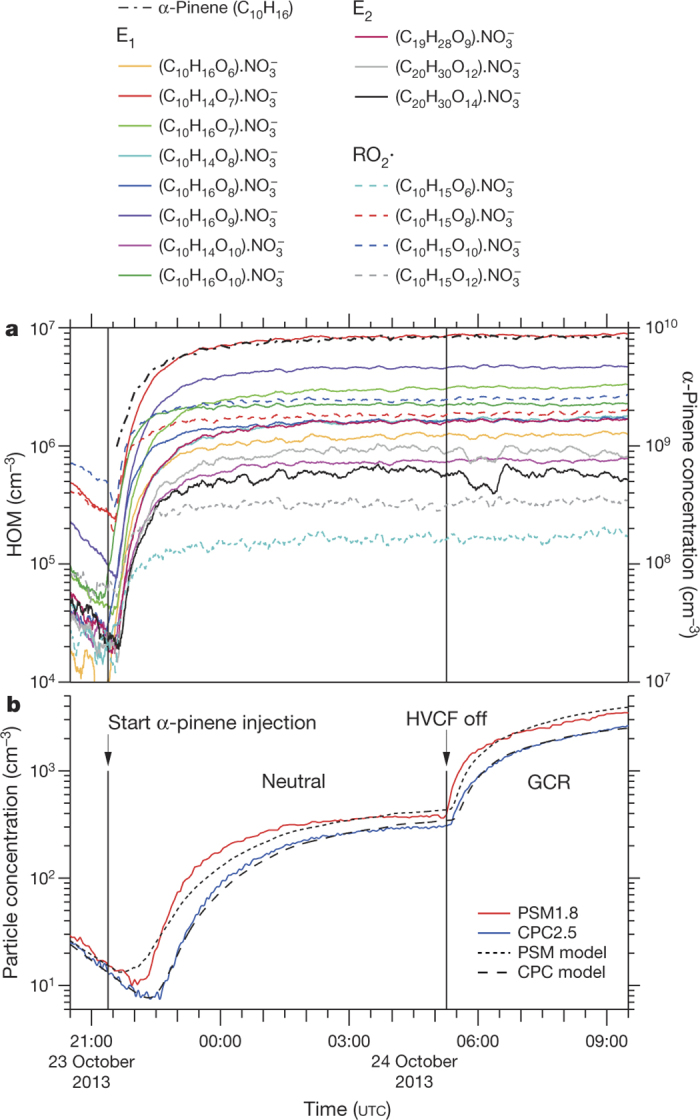
Evolution of HOMs and particles during a typical run. **a**, Evolution of selected HOM monomers (E_1_), dimers (E_2_) and peroxy radicals (RO_2_·) at 300 p.p.t.v. α-pinene, 33 p.p.b.v. O_3_, zero H_2_ or HONO, 38% relative humidity, 278 K and [H_2_SO_4_] < 5 × 10^4^ cm^−3^ (the same run as shown in Extended Data Fig. 4). The HOMs start to appear soon after the first injection of α-pinene into the chamber at 21:22, 23 October 2013. A HOM monomer is a highly oxygenated molecule derived from α-pinene (C_10_H_16_), and a HOM dimer is a covalently bound pair of monomers. Peroxy radicals are identified by an odd H number. The HOMs are charged with an  ion in the CI-APi-TOF mass spectrometer. The systematic scale uncertainty on the HOM concentrations is +80%/−45%. **b**, Evolution of the particle number concentrations measured in the PSM1.8 (red curve) and CPC2.5 (blue curve) particle counters. The high-voltage clearing field (HVCF) was switched off at 05:16, 24 October 2013, marking the transition from neutral (ion-free) to GCR conditions in the chamber. A sharp increase in the rate of particle formation is seen, due to ion-induced nucleation of pure biogenic particles. However, no change occurs in the HOM concentrations (**a**), because these are predominantly neutral gas-phase molecules. The dotted and dashed curves in **b** show the PSM1.8 and CPC2.5 distributions, respectively, simulated for this run with the AEROCLOUD kinetic model, which is used to derive the experimental nucleation rates (see Methods). PowerPoint slide

Within a few minutes of the initial exposure of α-pinene to O_3_ in the chamber, we detected gas-phase HOM monomers and dimers (Fig. 1a). Particles appeared shortly afterwards (Fig. 1b). HOM monomers (denoted E_1_) broadly comprise highly oxidized C_8–10_H_14,16_O_6–12_ species with an oxygen-to-carbon ratio (O/C) above about 0.6. HOM dimers (E_2_) are two covalently bound monomers (see below), which generally have lower oxygen-to-carbon ratios, but, almost certainly, a lower volatility. For the present study we define E_1_ (E_2_) to be the summed HOM peaks in the mass/charge range *m*/*z* = 235–424 Th (425–625 Th), where 1 Th = 1 Da/*e* and *e* is the elementary charge. This definition excludes peaks in the E_1_ mass band distinguished by an odd H number (C_10_H_15_O_6,8,10,12_), which we assign to the RO_2_· peroxy radical. These *m*/*z* values include a contribution of 62 Th due to the  ion from the CI-APi-TOF ionizer. We define the total HOMs as the sum RO_2_· + E_1_ + E_2_.

**Extended Data Figure 2:**
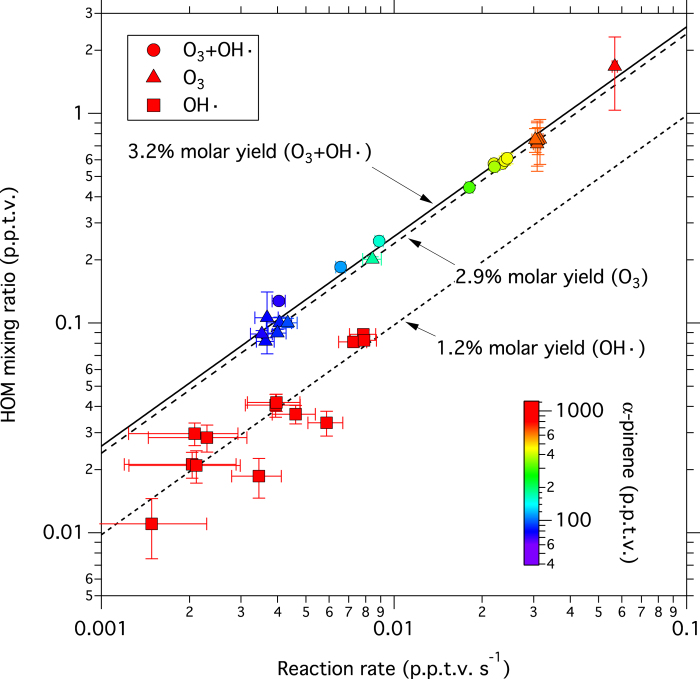
HOM yields versus α-pinene oxidation rates with O_3_ and OH. Total HOM mixing ratios versus α-pinene reaction rate with (i) O_3_ plus OH· (ozone without H_2_ scavenger; circles and solid line), (ii) O_3_ alone (ozone with 0.1% H_2_ scavenger; triangles and dashed line) and (iii) OH· alone (produced by ultraviolet photolysis of nitrous acid, HONO, in the absence of O_3_; squares and dotted line). The yields are shown for total HOMs = RO_2_· + E_1_ + E_2_. The experimental conditions are 38% relative humidity, 278 K and (i) 70–440 p.p.t.v. α-pinene, 21–35 p.p.b.v. O_3_, zero H_2_ or HONO, 0%–100% ultraviolet, (ii) 80–1,230 p.p.t.v. α-pinene, 21–35 p.p.b.v. O_3_, 0.1% H_2_, zero HONO, 0%–100% ultraviolet, and (iii) 840–910 p.p.t.v. α-pinene, zero O_3_ or H_2_, 0.5–3 p.p.b.v. HONO, 0%–100% ultraviolet. The bars indicate 1*σ* point-to-point errors. Overall systematic scale uncertainties of ±40% for the reaction rates and +80%/−45% for the HOM mixing ratios are not shown. The combined errors on the HOM molar yields for either ozonolysis or hydroxyl chemistry are +100%/−60% (±1*σ*).

We measure high HOM molar yields (Extended Data Fig. 2): approximately 1.2% per hydroxyl radical (OH·) reaction with α-pinene, 3.2% per ozone reaction with α-pinene, and 2.9% from pure ozonolysis. We find a high E_2_ yield from ozonolysis (10%–20% of total HOMs), but negligible E_2_ yield from hydroxyl-initiated oxidation. Neutral trimers are close to the detection limit of the CI-APi-TOF (below 0.1% of total HOMs). High yields of these same HOMs have previously been reported^6,16^, although our ozonolysis yields are less than half those of ref. 16. For our experiments, α-pinene was in the range 0.1–2 parts per billion by volume (p.p.b.v.), with 20–40 p.p.b.v. of O_3_. The OH· concentrations were (0.5–0.8) × 10^6^ cm^−3^ during ozonolysis experiments, and (0.4–2) × 10^5^ cm^−3^ during pure hydroxyl experiments with 0.5–3 p.p.b.v. of HONO.

**Extended Data Figure 3:**
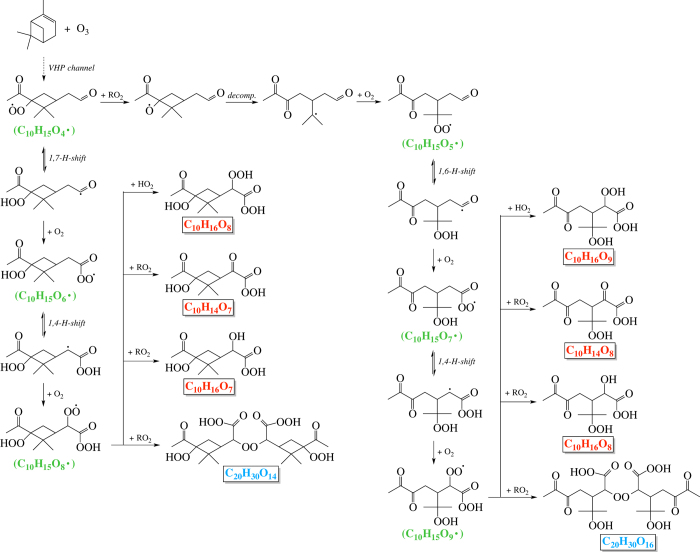
Proposed mechanism for the formation of the E_1_ and E_2_ surrogates via peroxy radical formation. The proposed scheme for the formation of the ELVOC monomer (C_10_H_14_O_7_) and dimer (C_20_H_30_O_14_) surrogates selected for quantum chemical calculations (Extended Data Fig. 7) is based on recently established autoxidation mechanisms for a series of cycloalkane + O_3_ systems^16,18,19,20,73,74^. Peroxy radicals in the figure are indicated by a green label, E_1_ by a red label and E_2_ by a blue label. Addition of ozone to the double bond of α-pinene produces two carbonyl-substituted Criegee biradicals. The energy-rich Criegee biradical is either collisionally stabilized, or isomerizes via 1,4-H-shift to a vinylhydroperoxide (VHP), which then decomposes to yield an OH· and an alkenoxy radical. The alkenoxy radical reacts with O_2_, leading to a peroxy radical, which is the potential precursor to a sequence of autoxidation reactions leading to the formation of HOMs^16^. Here the peroxy radical C_10_H_15_O_4_· is chosen as the starting point for HOM formation. The first intramolecular hydrogen abstraction is likely to take place at the aldehydic carbon from the opposite side of the peroxy group, although the rigid four-carbon-atom ring could hinder bending of the structure. For the *cis* configuration where the peroxy group and the aldehydic hydrogen are on the same side of the cyclobutyl ring, the 1,7-H shift rate is calculated^20^ to be 0.14 s^−1^, which initiates the autoxidation chemistry on a fast timescale compared to the HOM lifetime resulting from loss to the CLOUD chamber walls (about 900 s). The resultant acylic radical undergoes rapid O_2_ addition, leading to an -OOH functionalized peroxyacyl radical (C_10_H_15_O_6_·). The second intramolecular hydrogen abstraction is expected to proceed at the carbon atom in the α position of the peroxyacyl group via 1,4-H isomerization. The resultant C_10_H_15_O_8_· terminates by known reactions of peroxy radicals (HO_2_· or RO_2_· under the present experimental conditions), producing a spectrum of HOM monomers that includes the E_1_ surrogate, C_10_H_14_O_7_. The homogeneous recombination of two peroxy radicals via elimination of O_2_ produces the covalently bound dimer C_20_H_30_O_14_ chosen as the E_2_ surrogate. Alternatively, C_10_H_15_O_8_· can undergo further autoxidation, if sufficiently labile hydrogen atoms are available, leading to the observed closed-shell monomers with ≥9 O (Fig. 1). The self/cross-reaction of the C_10_H_15_O_4_· peroxy radical produces an alkoxy radical, which decomposes rapidly, leading to a carbonyl-functionalized peroxy radical (C_10_H_15_O_5_·). This peroxy radical is another potential starting structure for HOM formation. The carbon-ring-opening reaction pathway, while increasing the steric availability of the H atom, might be a slow step. The effective formation rate of the C=O-functionalized peroxy radical is calculated to be less than about 10^−3^ s^−1^, which is comparable to its wall deposition rate. The timescale with respect to the subsequent autoxidation reaction, on the other hand, is expected to be of the order of seconds, by analogy with that for branched-chain peroxy radicals^73^. The unbalanced sources and sinks potentially account for the low signals of peroxy radicals with odd oxygen numbers (for example, C_10_H_15_O_5_, C_10_H_15_O_7_ and C_10_H_15_O_9_). The autoxidation process of C_10_H_15_O_5_· is presumed to proceed by an autoxidative reaction pathway similar to that for C_10_H_15_O_4_·, eventually leading to the spectrum of HOM monomers and dimers observed in the CLOUD chamber. Except for the autoxidation channel, all the peroxy radicals are still subject to well-established reactions such as R(′)O_2_ + RO_2_/HO_2_, which are potentially important if the reaction rate is comparable to that for the autoxidation channel.

This remarkably fast production of HOMs is likely to proceed via an autoxidation mechanism involving peroxy radicals^16,18,19,20^ (Extended Data Fig. 3). There is simply insufficient time for oxidation to proceed in multiple steps through stable intermediate molecules. Here, initial ozonolysis of an α-pinene molecule proceeds via a Criegee intermediate and further steps to form an RO_2_· radical, followed by several repeated cycles of intramolecular H abstraction and O_2_ addition to re-form a new RO_2_· radical. We measure an RO_2_· fraction of total HOMs between 15% and 1% for HOMs from 0.1 p.p.t.v. to 10 p.p.t.v., respectively. A combination reaction of differently oxidized peroxy radicals explains the rapid high yield of covalently bound E_2_. The negligible E_2_ yield from hydroxyl-initiated oxidation could result from additional NO_*x*_ chemistry that terminates the peroxy radicals before they can combine. Our theoretical calculations further indicate that E_2_ must be covalently bound because the neutral molecular cluster formed from two monomers (denoted E_1_.E_1_) is expected to be unstable (see below).

We measured nucleation rates under neutral (*J*_n_), Galactic cosmic ray (GCR; *J*_gcr_) and *π*^+^ beam (*J*_*π*_) conditions, corresponding to ion-pair concentrations of around 0 cm^−3^, 700 cm^−3^ and 3,000 cm^−3^, respectively. This range spans atmospheric ion concentrations between ground level and 15-km altitude. The nucleation rate *J*_n_ describes the neutral rate alone, whereas *J*_gcr_ and *J*_*π*_ describe the sum of the neutral and ion-induced rates, *J*_n_ + *J*_iin_. We determine the nucleation rates at 1.7-nm mobility diameter, at which size a particle is generally considered to be stable against evaporation. To determine the nucleation rates, we fit the time-dependent particle concentrations with a numerical model that treats particle nucleation and growth kinetically at the molecular level (an example is shown in Fig. 1b; see Methods for further details).

**Extended Data Figure 4:**
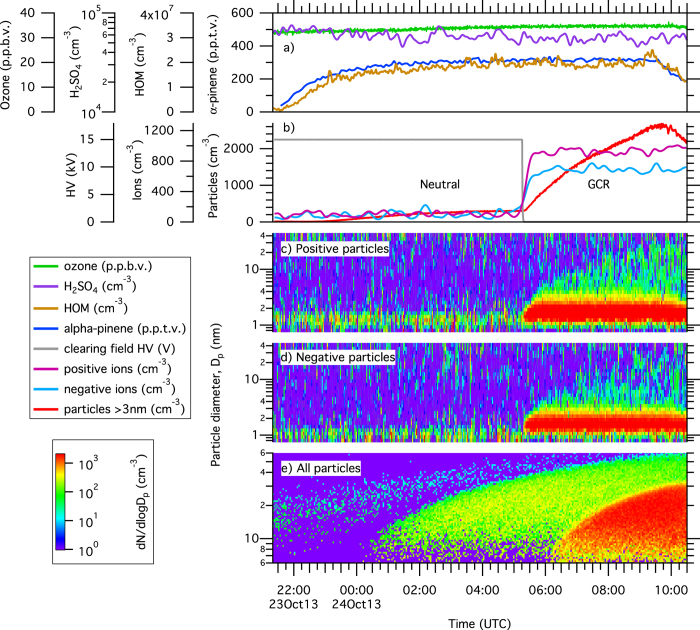
Typical nucleation run sequence. Example of a typical measurement sequence of the neutral and GCR nucleation rates as a function of coordinated universal time (utc), at zero H_2_ or HONO, 38% relative humidity and 278 K. **a**, The run began at 21:22, 23 October 2013, by starting the α-pinene flow into the chamber to reach a chosen equilibrium value near 300 p.p.t.v., which produced an equilibrium total HOMs concentration near 2 × 10^7^ cm^−3^ (0.8 p.p.t.v.). **b**, Particles (red curve) formed at a slow rate in the chamber without ions present (‘neutral’ conditions). The clearing field high voltage (HV) was turned off at 05:16, 24 October 2013, and the subsequent presence of ions in the chamber from GCRs caused a sharp increase in the particle formation rate by about one order of magnitude (as seen by the increase in the gradient of the red curve). The nucleation rates are measured under constant gas conditions in the period before (*J*_n_ = 0.14 cm^−3^ s^−1^) and after (*J*_gcr_ = 3.3 cm^−3^ s^−1^) turning off the clearing field high voltage. **c**, **d**, Ion-induced nucleation is observed both for positive (**c**) and negative (**d**) charged particles, followed by rapid particle growth to sizes above 10 nm. **e**, The nucleated particles grew over a period of several hours to diameters approaching 50 nm, where they begin to constitute cloud condensation nuclei. A sharp increase in the formation rate of particles above the SMPS detection threshold of 5 nm can be seen when GCR ions are present. The colour scale in **c**–**e** indicates d*N*/dlog(*D*_p_), where *N* (in cm^−3^) is the particle number concentration and *D*_p_ (in nm) is the particle diameter. The concentrations of ozone and contaminant H_2_SO_4_ were essentially constant during the run, which ended at 09:30 when the α-pinene flow to the chamber was turned off. The H_2_SO_4_ measurement near 5 × 10^4^ cm^−3^ corresponds to the instrumental background level of the CI-APi-TOF mass spectrometer and so represents an upper limit on the actual concentration. Further characteristics of this run can be seen in Fig. 1.

**Extended Data Figure 5:**
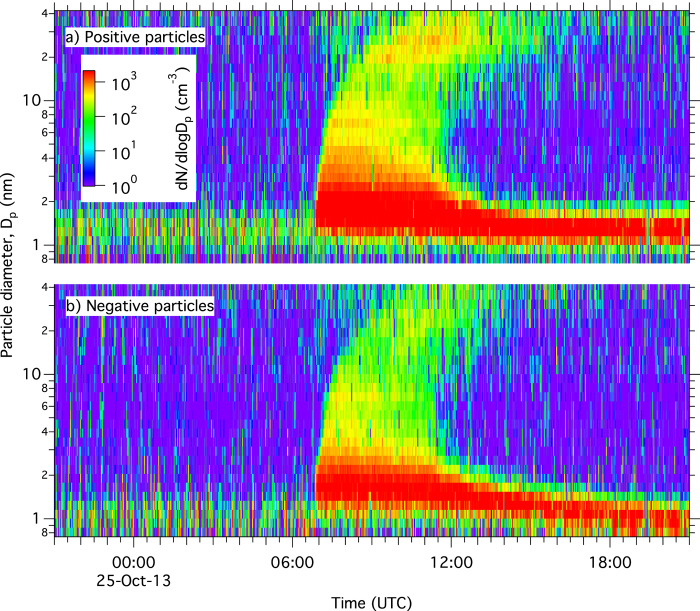
Ion-induced nucleation event without H_2_SO_4_, measured in the NAIS. **a**, **b**, Example of a nucleation event showing the growth versus time of positive (**a**) and negative (**b**) charged particles at 530 p.p.t.v. α-pinene, 35 p.p.b.v. O_3_, zero H_2_ or HONO, 3.4 × 10^7^ cm^−3^ HOM, 38% relative humidity, 278 K and [H_2_SO_4_] < 5 × 10^4^ cm^−3^. The colour scale shows the concentration of ions and charged particles. The clearing field high voltage was turned off at 06:48, marking the start of GCR ionization conditions in the chamber, and the α-pinene flow into the chamber was stopped at 10:52. Ion-induced nucleation can be seen for positive and negative charged particles, followed by rapid growth to sizes above 10 nm. Ion–ion recombination progressively neutralizes the charged particles as they grow, but some reappear at larger sizes, owing to diffusion charging.

A typical run sequence (Extended Data Fig. 4) begins by establishing ion-free conditions with a high-voltage clearing field and introducing α-pinene to the chamber, where it mixes with ozone. Particles then start to form and, after measuring *J*_n_ at steady-state α-pinene concentration, we turn off the high voltage and measure *J*_gcr_ under otherwise identical chamber conditions. A sharp enhancement of particle formation is seen when the high voltage was turned off (Extended Data Fig. 4b, e), due to ion-induced nucleation of both charge signs (Extended Data Figs 4c, d and 5).

**Figure 2:**
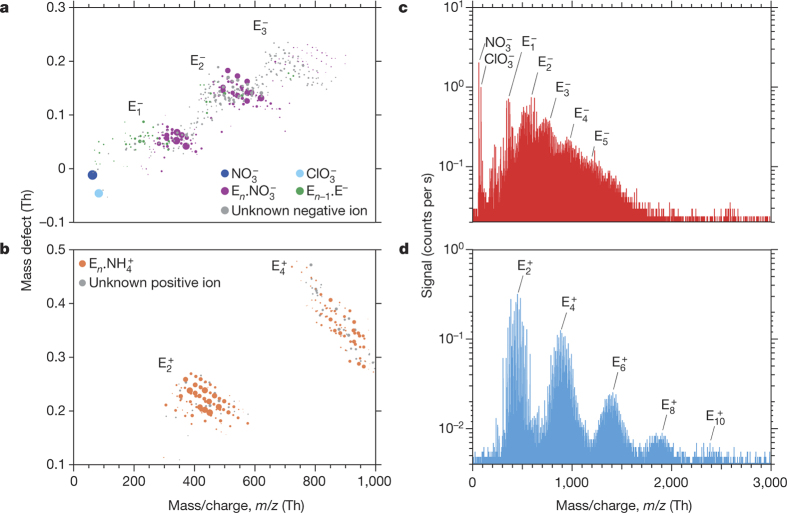
Molecular composition and mass spectra of charged clusters during GCR nucleation events without sulfuric acid. **a**, **b**, Cluster mass defect (difference from integer mass) versus *m*/*z* of negatively (**a**) and positively (**b**) charged clusters measured with the APi-TOF at 240 p.p.t.v. α-pinene, 34 p.p.b.v. O_3_, zero H_2_ or HONO, 38% relative humidity, 278 K and [H_2_SO_4_] below the detection limit (5 × 10^4^ cm^−3^). The values of *J*_gcr_ and total HOMs concentration are, respectively, 3.4 cm^−3^ s^−1^ and 1.7 × 10^7^ cm^−3^ (**a**), and 3.3 cm^−3^ s^−1^ and 2.4 × 10^7^ cm^−3^ (**b**). The mass bands are labelled according to the number of HOM monomer units in the cluster, E_*n*_. Each circle represents a distinct molecular composition and its area represents the counts per second. The most highly oxidized compounds are located at the lower right-hand edge of each band. The dark blue circle represents  ions; the light blue circle represents  ions. Clusters with fully identified molecular composition are coloured according to their core ion: purple (), green (E^−^) or orange (). Grey circles are unidentified clusters. **c**, **d**, Mass spectra from the same events for negative (**c**) and positive (**d**) clusters up to *m*/*z* = 3,000 Th. A particle of 1.7-nm mobility diameter has a mass of about 1,200 Th. The ‘Nessie’ plot (**d**) shows that positive-ion-induced nucleation involves HOM dimers alone (E_1_. clusters are not seen owing to instrument tuning). The decreasing signal amplitude at larger masses is due to the lower concentration and decreasing detection efficiency of the APi-TOF mass spectrometer (the efficiency versus *m*/*z* depends on the instrument tune and polarity). PowerPoint slide

Figure 2 shows the molecular composition and mass spectra of negatively and positively charged ions, monomers, dimers and clusters during ion-induced nucleation events. The dominant core ions in the clusters are identified as ,  and E^−^. Here E^−^ is inferred for negatively charged ions or clusters that contain only C, H and O; the E^−^ ion corresponds to a HOM of high gas-phase acidity. In contrast to negative clusters, the positive clusters nucleate only with dimers, producing distinct mass bands that are detected up to E_10_ in the APi-TOF (Fig. 2c, d). This indicates the importance of dimers for pure biogenic nucleation. Dimers are expected to be less volatile than monomers, owing primarily to higher molecular weight, but also to additional functional groups. Our previously described definition for neutral gas-phase HOMs encompasses compounds with a wide range of low volatilities^19,21^, of which only a subset drive nucleation (ELVOCs, which comprise about 36% of measured total HOMs^21^). From the strong ion enhancement of nucleation we conclude that the APi-TOF mass peaks above the dimer in Fig. 2 are clusters of ELVOC monomers and dimers. Although we can precisely determine their molecular composition (C_*x*_H_*y*_O_*z*_), we can only infer their specific structure and functional groups.

**Figure 3:**
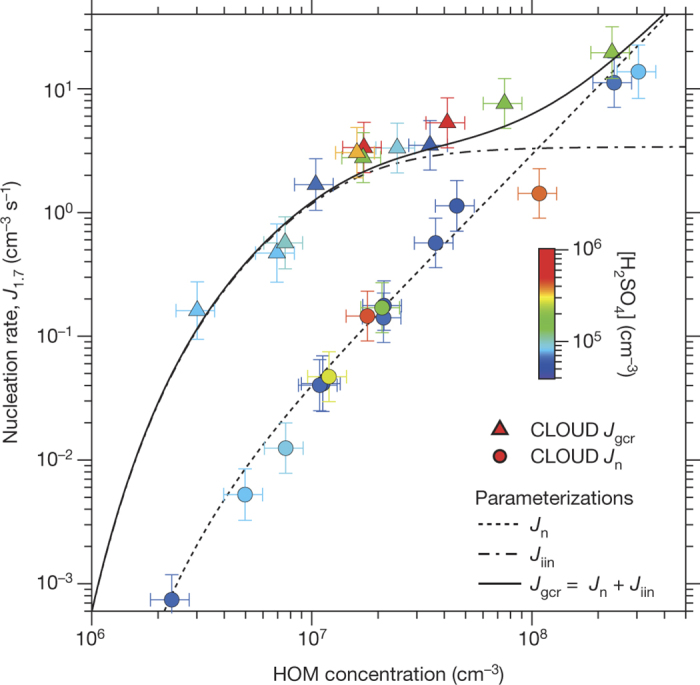
Pure biogenic nucleation rates versus HOM concentration. Neutral (*J*_n_; circles) and GCR (*J*_gcr_; triangles) nucleation rates versus total HOMs concentration (RO_2_· + E_1_ + E_2_). The fraction of total HOMs that participate in nucleation (ELVOCs) is about 36% (ref. 21). The experimental conditions are 10–1,300 p.p.t.v. α-pinene (for measurements below *J*_1.7_ = 10 cm^−3^ s^−1^), 30–35 p.p.b.v. O_3_, zero H_2_ or HONO, 38% relative humidity, 278 K and <8 × 10^5^ cm^−3^ H_2_SO_4_. The colour scale shows [H_2_SO_4_]; purple and blue points correspond to contaminant level (below the detection threshold); other colours correspond to measurements after SO_2_ was added to the chamber. The fitted curves show parameterizations (described in Methods) for *J*_n_ (dashed), *J*_gcr_ (solid) and ion-induced nucleation (*J*_iin_ = *J*_gcr_ − *J*_n_; dot-dashed). The *J*_iin_ parameterization assumes that the nucleation rate falls steeply at HOM concentrations below the experimental measurements, following a similar slope to that for *J*_n_. The bars indicate 1*σ* total errors, although the overall systematic scale uncertainty of +80%/−45% on the HOM concentration is not shown. PowerPoint slide

**Extended Data Figure 6:**
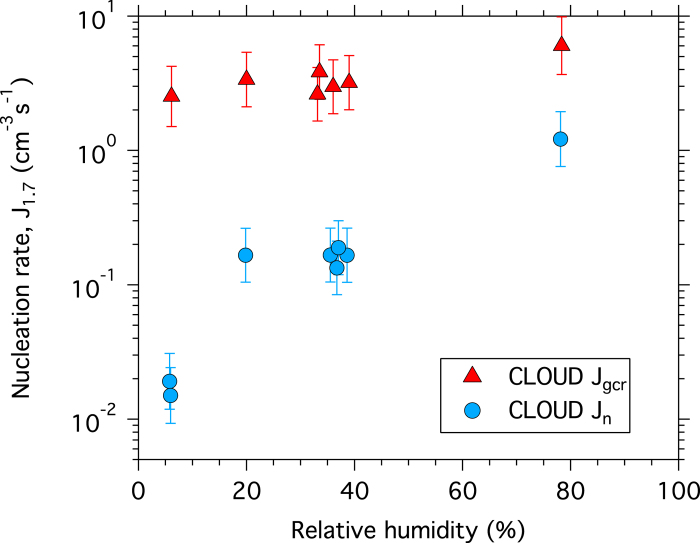
Nucleation rates versus relative humidity. Neutral (*J*_n_; circles) and GCR (*J*_gcr_; triangles) nucleation rates versus relative humidity. The experimental conditions are 250–800 p.p.t.v. α-pinene, 30–35 p.p.b.v. O_3_, zero H_2_ or HONO, (1.1−2.9) × 10^7^ cm^−3^ HOM, 278 K and (0.5−1.5) × 10^5^ cm^−3^ H_2_SO_4_. All measurements have been corrected to the same total HOMs concentration (2.05 × 10^7^ cm^−3^) using the curves shown in Fig. 3. The bars indicate 1*σ* total errors, although these are not shown in the *x* direction because they are smaller than the symbols.

We show the experimental neutral and GCR nucleation rates in Fig. 3 over the total HOMs range 0.1–10 p.p.t.v., which spans the range of atmospheric interest. Below 1 p.p.t.v. HOM, ionization at ground-level GCR intensities enhances the nucleation rate by between one and two orders of magnitude compared with neutral nucleation. At higher concentrations, the neutral and GCR nucleation rates converge because the ion-induced rate, *J*_iin_, reaches the limit set by the GCR total ion production rate (3.4 cm^−3^ s^−1^). Positive and negative clusters nucleate at comparable rates (an example is shown in Extended Data Fig. 5). Relative humidity has little effect on *J*_gcr_ over the range 6%–80% relative humidity, whereas *J*_n_ increases substantially at higher relative humidity (Extended Data Fig. 6).

The large GCR enhancement indicates that biogenic molecular clusters are relatively unstable unless an ion is present. A charged cluster is also likely to experience higher collision rates with HOMs because they are expected to have high electric polarizability and, depending on their structure, large dipole moments. We further investigated the dependence on ion species by adding small amounts of SO_2_ to the chamber, up to around 1,000 p.p.t.v. When [H_2_SO_4_] exceeds about 1 × 10^5^ cm^−3^, the major negative ion species shift to ,  and  (Extended Data Fig. 1c), owing to their lower proton affinity (higher gas-phase acidity) than contaminant compounds. However, the nucleation rates with sulfur ion species remain unchanged (Fig. 3). Taken together, our observations therefore show that ubiquitous ion species can stabilize embryonic biogenic clusters. However, we do not observe chlorine in nucleating clusters, even though contaminant chlorine ion species are present (Fig. 2 and Extended Data Fig. 1), which indicates that not all ions have a suitable chemical structure to bond strongly with the oxidized organic compounds^22^.

**Figure 4:**
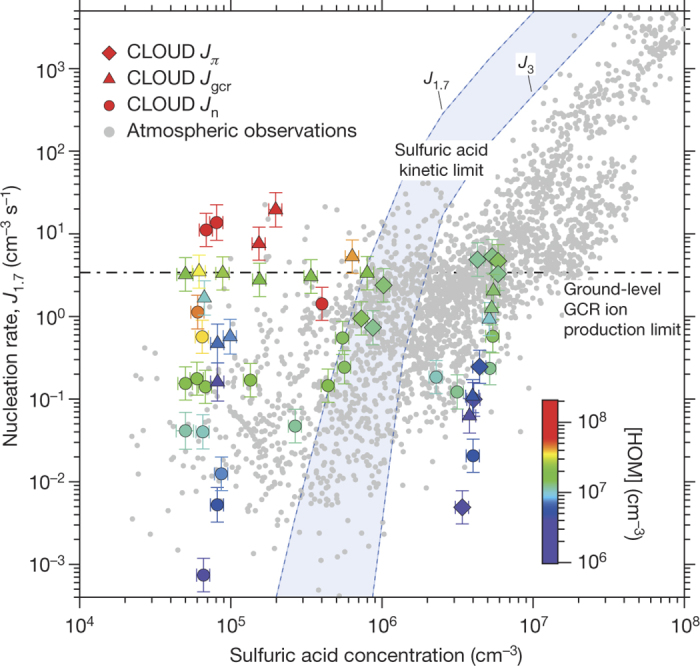
Experimental and atmospheric nucleation rates versus H_2_SO_4_ concentration. CLOUD measurements of the neutral (*J*_n_; circles), GCR (*J*_gcr_; triangles) and *π* beam (*J*_*π*_; diamonds) biogenic nucleation rates at 1.7 nm (*J*_1.7_) versus [H_2_SO_4_]. The CLOUD experimental conditions are 10–1,300 p.p.t.v. α-pinene (for measurements below *J*_1.7_ = 10 cm^−3^ s^−1^), 25–35 p.p.b.v. O_3_, zero H_2_ or HONO, 20%–40% relative humidity and 278 K. Measurements below 1 × 10^5^ cm^−3^ for [H_2_SO_4_] are near to the detection limit of the CI-APi-TOF and should be considered as upper-estimates (to avoid overlap, some data points at the H_2_SO_4_ detection limit are displaced by up to 1 × 10^4^ cm^−3^). The total HOMs concentration from α-pinene oxidation is indicated by the colour scale. Observations of particle formation in the atmospheric boundary layer (mainly at 3-nm threshold size) are indicated by small grey circles^3,4,23,24^. Following convention, the H_2_SO_4_ concentration refers to monomers alone; that is, H_2_SO_4_ bound in molecular clusters is not included. The kinetic upper limit on sulfuric acid nucleation is indicated by the blue band, which is bounded by dashed lines indicating *J*_1.7_ and *J*_3_. This band assumes the CLOUD condensation sink, which is comparable to that of a pristine atmosphere. The upper limit on *J*_iin_ from the GCR ion-pair production rate at ground level is indicated by the dot-dashed line. The bars indicate 1*σ* total errors, although the overall +50%/−33% systematic scale uncertainty on [H_2_SO_4_] is not shown. PowerPoint slide

Figure 4 shows the CLOUD biogenic nucleation rates extended to [H_2_SO_4_] = 6 × 10^6^ cm^−3^ and compared with atmospheric boundary-layer observations^3,4,23,24^. Biogenic nucleation rates show no significant dependence on sulfuric acid concentration over this range (that is, within the experimental measurement errors, the nucleation rate is consistent with zero dependency on sulfuric acid concentration). This finding sharply contrasts with base-stabilized nucleation of sulfuric acid in the presence of ammonia^9^ or amines^10^, where nucleation rates at 1.7 nm show a steep dependency on [H_2_SO_4_] above 10^6^ cm^−3^. Comparison of the atmospheric observations (Fig. 4) with our measurements therefore suggests that nucleation in the lower atmosphere may involve a mixture of two distinct mechanisms. The first, which is more important in polluted environments, involves nucleation of sulfuric acid and water together with a combination of amines or ammonia with oxidized organics, and has a strong dependence on sulfuric acid. The second, which is more important in pristine environments, involves nucleation of pure organic particles and depends on only oxidized organics and ions.

**Extended Data Figure 7:**
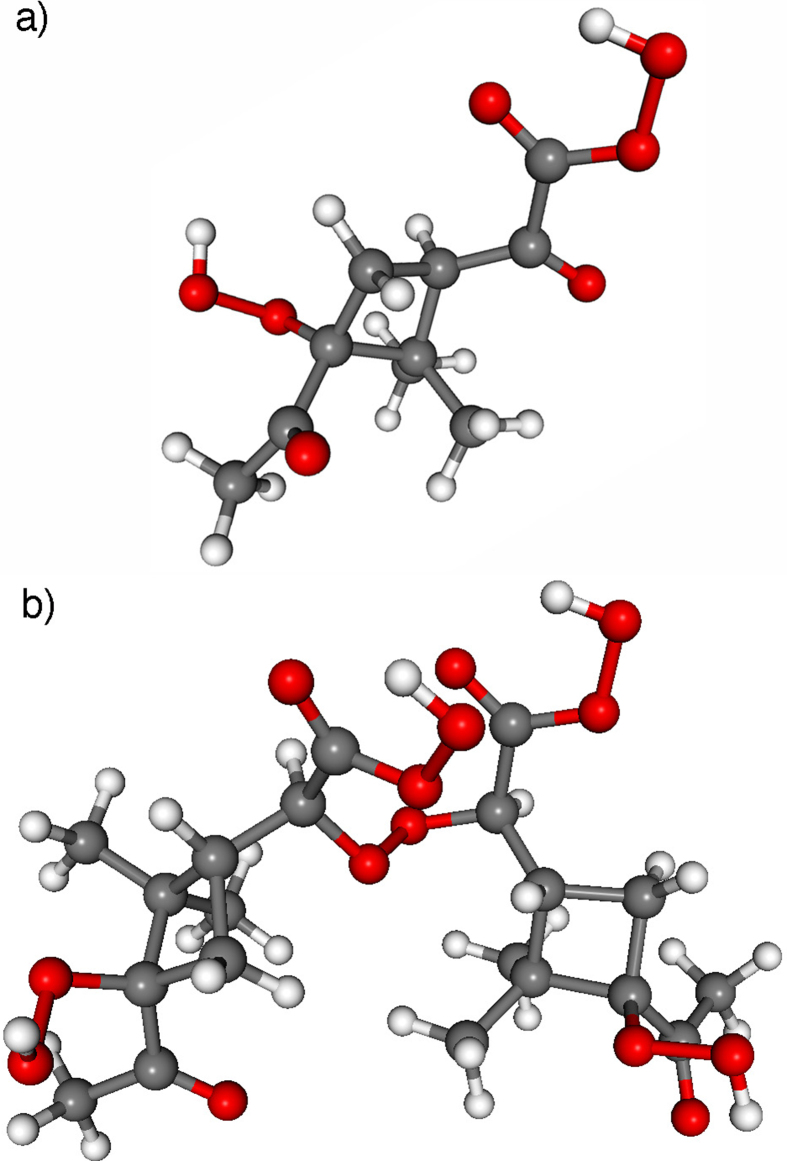
Surrogate molecules chosen for quantum chemical calculations. **a**, **b**, Structures of the surrogate molecules chosen for quantum chemical calculations to represent the ELVOC monomer, E_1_, C_10_H_14_O_7_ (**a**) and the covalently bound dimer, E_2_, C_20_H_30_O_14_ (**b**). Grey spheres represent carbon atoms, red are oxygen atoms and white are hydrogen atoms. We show their proposed formation mechanisms in Extended Data Fig. 3.

**Extended Data Figure 8:**
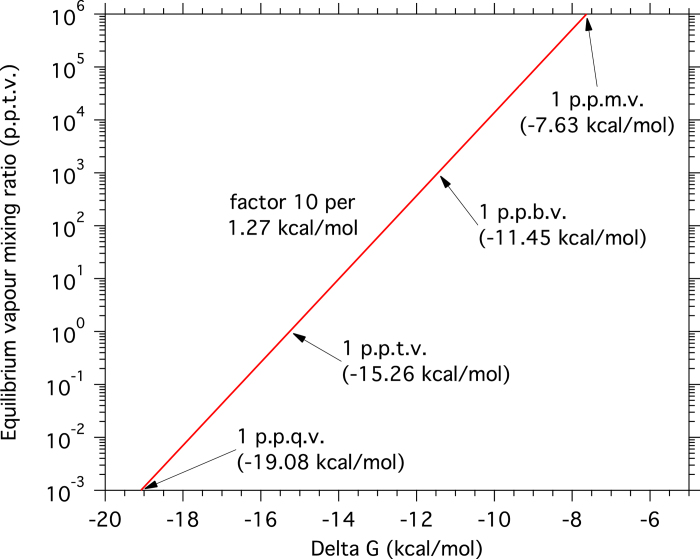
Relationship between cluster formation energies and equilibrium evaporation/condensation rates. Estimated ELVOC vapour mixing ratios versus the Δ*G*_278 K_ at which the condensation and evaporation rates of the cluster at 278 K are in equilibrium^68,69^. For example, a formation free energy of −15.3 kcal mol^−1^ corresponds to equal rates for particle evaporation and vapour condensation at 278 K and 1 p.p.t.v. ELVOC vapour mixing ratio (2.6 × 10^7^cm^−3^). The evaporation rate increases by a factor of 10 for each 1.27 kcal mol^−1^ reduction of the cluster formation energy.

To gain further insight into the stability of initial neutral and charged clusters of highly oxidized biogenic molecules, we calculated their Gibbs free energies of formation, Δ*G*, using quantum chemical methods (see Methods). For this study we chose C_10_H_14_O_7_ and C_20_H_30_O_14_ as E_1_ and E_2_ surrogates, respectively (Extended Data Fig. 7). We observe these compounds both in the gas (Fig. 1) and particle phases in the CLOUD chamber. We show proposed formation mechanisms and structures^19,20^ in Extended Data Fig. 3. Our calculations, summarized in Extended Data Table 1 and Extended Data Fig. 8, confirm that ELVOC clusters formed with an , ,  or  ion are expected to be stable (that is, their growth rate exceeds the evaporation rate) at around 0.1 p.p.t.v. ELVOC, or below. In contrast, the initial neutral clusters are weakly bound and so neutral nucleation is expected to be weaker. Although limited to a single surrogate pair, our theoretical calculations thus provide independent support for the experimental measurements.

Comparisons with atmospheric observations should be considered as preliminary because our measurements were made at only one temperature, with a single monoterpene, in the absence of isoprene and mostly in the absence of NO_*x*_, which can influence HOM yields. Nevertheless, our results may provide fresh insights into several seemingly disparate phenomena associated with low atmospheric concentrations of sulfuric acid. First, pure HOM nucleation could provide a mechanism to account for nucleation-mode particles observed at night-time, under low-[H_2_SO_4_] conditions^25,26^. Second, although observations are rare, nucleation-mode particles are seen in the Amazon^27^, where SO_2_ levels are extremely low (20–30 p.p.t.v.). Peak particle concentrations often occur at sunrise and sunset^27^, and appear to be associated with rain, which reduces the aerosol condensation sink and may generate high ion concentrations by evaporation of charged droplets at the Rayleigh limit. Third, pure biogenic nucleation could explain new particle formation observed in the upper troposphere in cloud outflows depleted of SO_2_, such as over the Amazon^27,28,29^. Low-solubility biogenic precursor vapours can be efficiently convected inside clouds to high altitudes where HOMs will form in the cloud outflows on exposure to oxidants, and nucleation is likely to be enhanced by the low temperatures. Fourth, since high HOM yields are also found from other organic compounds with an endocyclic double bond such as cyclohexene^16^, pure HOM nucleation involving anthropogenic organic precursors could be expected when [H_2_SO_4_] is low^30^. Finally, ion-induced pure biogenic nucleation might shed new light on the long-standing question of a physical mechanism for solar-climate variability in the pristine pre-industrial climate^31,32^.

Direct observational evidence of pure biogenic nucleation has not been reported so far, owing to atmospheric pollution or lack of suitable instrumentation. The pure biogenic mechanism is likely to dominate nucleation in pristine terrestrial regions such as tropical rainforests or at higher altitudes above forests in convective cloud outflows. Pure biogenic nucleation might also take place over forested areas at high northern latitudes during periods of especially low pollution. Identification of pure biogenic nucleation in the atmosphere will require simultaneous measurements with several newly developed mass spectrometers, APi-TOF (for molecular composition of ions and nucleating charged clusters) and CI-APi-TOF (gas-phase HOMs and H_2_SO_4_), together with standard instruments such as low-threshold particle counters, PTR-TOF (precursor organic vapours) and NAIS (size spectra of ions and charged particles).

In summary, we find that highly oxidized organic compounds play a role in atmospheric particle nucleation comparable to that of sulfuric acid; together with a suitable stabilizing agent, each has sufficiently low volatility to form new particles in the lower atmosphere at vapour concentrations near 10^7^ cm^−3^. The stabilizing agent for pure biogenic particles is a suitable ion, whereas for sulfuric acid particles the stabilizing agents are amines, or ammonia with oxidized organics. Ion-induced nucleation of pure biogenic particles may have important consequences for pristine climates because it provides a mechanism by which nature produces particles without pollution. This could raise the baseline aerosol state of the pristine pre-industrial atmosphere and so could reduce the estimated anthropogenic radiative forcing from increased aerosol-cloud albedo over the industrial period.

## Methods

### Overview of the CLOUD facility

The CLOUD experiment at CERN is designed to study the effects of cosmic rays on aerosols, cloud droplets and ice particles, under precisely controlled laboratory conditions. The 3-m-diameter stainless-steel CLOUD chamber and its gas system have been built to the highest technical standards of cleanliness and performance. The CLOUD chamber is periodically cleaned by rinsing the walls with ultra-pure water, followed by heating to 373 K and flushing at a high rate with humidified synthetic air and elevated ozone (several parts per million by volume). Contaminant levels of condensable vapours are in the sub-p.p.t.v. range. The high cleanliness of the chamber, together with its large volume (26.1 m^3^) and highly stable operating conditions, allows particle formation to be studied under atmospheric conditions at nucleation rates between about 0.001 cm^−3^ s^−1^ and 100 cm^−3^ s^−1^. The loss rate of condensable vapours and particles onto the chamber walls is comparable to the ambient condensation sink of the pristine boundary layer.

Ion production in the chamber can be controlled using an internal electric clearing field (which creates an ion-free environment), GCRs or an adjustable *π*^+^ beam^9,33^ from the CERN Proton Synchrotron. The *π*^+^ beam is de-focused to a transverse size of about 1.5 m × 1.5 m when it passes through the CLOUD chamber. With the electric field set to zero, the equilibrium ion-pair concentration in the chamber due to GCRs is around 700 cm^−3^. With the *π*^+^ beam, this can be increased to any value up to about 3,000 cm^−3^. Hence, ion concentrations corresponding to any altitude in the troposphere can be generated in the CLOUD chamber.

The experiment has precise control of the trace vapours inside the chamber and also of the environmental temperature between 300 K and 203 K. Uniform mixing is achieved with magnetically coupled stainless-steel fans mounted at the top and bottom of the chamber. The characteristic gas mixing time in the chamber is a few minutes, depending on the fan speeds. Photochemical processes are initiated by illumination with an ultraviolet fibre-optic system, providing highly stable gas-phase reactions with a precise start time. The contents of the chamber are continuously analysed by instruments connected to sampling probes that project into the chamber. The sampling analysers are tailored for each experimental campaign, but typically comprise around 30–35 instruments, of which up to 10 are mass spectrometers.

### Summary of analysing instruments

For the results reported here, the analysing instruments attached to the chamber included a chemical ionization mass spectrometer (CIMS) for H_2_SO_4_ concentration^34^; an atmospheric pressure interface time-of-flight (APi-TOF; Aerodyne Research Inc. and Tofwerk AG)^35^ mass spectrometer for molecular composition of positively or negatively charged ions and clusters; two chemical ionization atmospheric pressure interface time-of-flight (CI-APi-TOF; Aerodyne Research Inc. and Tofwerk AG)^36,37^ mass spectrometers for molecular composition and concentration of neutral gas-phase H_2_SO_4_ and HOMs; a proton transfer reaction time-of-flight (PTR-TOF; Ionicon Analytik GmbH)^38^ mass spectrometer for organic vapours; a neutral cluster and air ion spectrometer (NAIS; Airel Ltd)^39^ for concentrations of positive ions, negative ions and charged clusters in the range 1–40 nm; a nano-radial differential mobility analyser (nRDMA)^40^ and a nano scanning mobility particle sizer (nano-SMPS) for particle size spectra; and several condensation particle counters (CPCs) with 50% detection efficiency thresholds between 1 nm and 4 nm: two Airmodus A09 particle size magnifiers, PSM^41^, (one fixed-threshold and the other scanning), two diethylene glycol CPCs, DEG-CPC^42,43^, a butanol TSI 3776 CPC and a water TSI 3786 CPC (TSI Inc.).

Additional gas analysers included dew-point sensors (EdgeTech), sulfur dioxide (Thermo Fisher Scientific, Inc. 42i-TLE) and ozone (Thermo Environmental Instruments TEI 49C). For certain tests, HONO vapour was supplied to the chamber and photolysed with ultraviolet light to produce OH· in the absence of O_3_. The gaseous HONO was generated by continual mixing of H_2_SO_4_ with NaNO_2_ (ref. 44) in a specially designed stainless-steel reactor, and then steadily flowed into the chamber. The HONO analyser involved a specially designed probe that passed samples of air from the chamber through a solution of H_2_SO_4_ and sulfanilamide, which was then analysed online with a long path absorption photometer (LOPAP)^45^.

### Determination of the nucleation and growth rates

The nucleation rates (in cm^–3^ s^–1^) were measured under neutral (*J*_n_), ground-level GCR (*J*_gcr_) and *π*^+^ beam (*J*_*π*_) conditions. Neutral nucleation rates are measured with the clearing field electrodes set to ±30 kV, which establishes an electric field of about 20 kV m^−1^ in the chamber. This completely suppresses ion-induced nucleation because, under these conditions, small ions or molecular clusters are swept from the chamber in about 1 s. Because all of the nucleation and growth processes under consideration take place on substantially longer timescales, neutral nucleation rates can be measured with zero background from ion-induced nucleation. For GCR and *π*^+^ beam conditions, the electric field was set to zero, leading to equilibrium ion-pair concentrations around 700 cm^−3^ and 3,000 cm^−3^, respectively. The nucleation rate *J*_n_ measures the neutral rate alone, whereas *J*_gcr_ and *J*_*π*_ measure the sum of the neutral and ion-induced nucleation rates, *J*_n_ + *J*_iin_.

The nucleation rates reported here were obtained primarily with the Airmodus scanning PSM at 1.8-nm threshold (PSM1.8) and the TSI 3776 CPC (CPC2.5), nominally 2.5-nm threshold, but measured at 3.2-nm threshold with WO_*x*_ particles^46^. The nucleation rates *J*_1.7_ are determined at 1.7-nm mobility diameter (1.4-nm mass diameter), at which size a particle is normally considered to be above its critical size and, therefore, thermodynamically stable. The critical size corresponds to the cluster size at which the evaporation and growth rates are equal. It varies with temperature, chemical species, charge and vapour concentrations, and may even be absent when evaporation rates are highly suppressed, such as for sulfuric acid–dimethylamine clusters^10,37^. Our measurements indicate that the smallest neutral HOM clusters are relatively unstable; therefore, 1.7 nm, which is equivalent to around 5 HOM monomer units, is a reasonable size at which to derive the experimental nucleation rates.

### AEROCLOUD model

To determine nucleation rates at 1.7 nm, the time-dependent particle concentrations measured with the PSM1.8 and CPC2.5 are fitted with a simplified numerical model (AEROCLOUD) that treats particle nucleation and growth kinetically at the molecular level. The model uses HOM monomer, HOM dimer and H_2_SO_4_ production rates derived from the CI-APi-TOF experimental data. The measured HOM production rates are scaled by a factor of 1.8 to match the observed particle appearance times and growth rates. This scaling results in good agreement of the model with the experimental data over the full experimental range of HOM concentrations. The scaling factor is within the systematic measurement uncertainty of the CI-APi-TOF, and could arise if a nitrate CI-APi-TOF does not detect all the HOMs that contribute to particle growth.

Primary ions from GCRs are generated in the model at the known rate of *q* = 1.7 ion pairs per cubic centimetre per second. A fixed parameter of the model, *f*_c_, accounts for the charge sign asymmetry due to differences in the diffusional loss rates of positive and negative primary ions to the chamber walls:The parameter *f*_c_ is determined by the experimentally measured positive and negative ion concentrations in the NAIS to have the value 0.52.

Molecules and particles collide kinetically, and cluster with each other. The model uses a reduced clustering probability (termed a ‘sticking probability’ below) to account for unstable small clusters, rather than allowing clusters to evaporate once they have formed. This greatly increases the speed of the computation. If the particle formed by a collision exceeds a certain size (corresponding to around 1.7-nm mobility diameter for pure biogenic clusters; see below), then it is assumed to be effectively stable and subsequently grows at near the kinetic limit. The particle growth rate between the PSM1.8 and CPC2.5 is therefore implicitly treated in the model essentially as kinetically limited growth by particle coagulation plus HOM and H_2_SO_4_ vapour condensation. Particles grow through size bins that are linearly spaced for small sizes and logarithmically spaced from about 2 nm to a maximum size of 400 nm. The time-steps for clustering processes range from 0.9 s to 10 s, depending on the conditions of the experimental run under analysis. The time-step is 10 s for all other processes (for example, updates of gas concentrations, high-voltage clearing-field changes, fan changes, and particle losses due to dilution of the chamber contents or diffusion to the walls). The density of the pure HOM clusters is fixed at 1.3 g cm^−3^, and at 1.85 g cm^−3^ for a pure H_2_SO_4_ cluster.

For neutral–neutral collisions, the number of particles in size bins 1 and 2 that coagulate in a time interval Δ*t* to produce a particle of mass *m*_12_ is:where *K*_00_ is the neutral–neutral collision kernel, *n*_1_, *n*_2_ and *n*_12_ are the particle number concentrations, and *V*_12_ is the van der Waals enhancement factor (see below). The neutral–neutral sticking probability for pure biogenic particles, , is:where *C*_B_ and *S*_B_ are free parameters. The parameter *C*_B_ effectively defines the threshold mass of stable clusters because the sticking probability  when *C*_*B*_ = *m*_12_, whereas the parameter *S*_B_ controls the sharpness of the threshold. The sticking probability for collisions where at least one particle is mainly sulfuric acid is similarly defined as:where *C*_A_ and *S*_A_ are free parameters.

The neutral–neutral collision kernel, *K*_00_, in equation (1) is the Fuchs form of the Brownian coagulation coefficient^47,48^. The van der Waals enhancement factor is the modification to Fuchs theory due to Sceats^49^, as described in ref. 50, for a Knudsen number in the kinetic (free molecular) regime. The enhancement factor is:where the reduced Hamaker constant, *A*′, is:where *r*_1,2_ are the particle radii, *A* = 6.4 × 10^−20^ J (the Hamaker constant for sulfuric acid^50^), *b*_0_ = 0.0151, *b*_1_ = −0.186, *b*_2_ = −0.0163, *k* is the Boltzmann constant and *T* is temperature. The same Hamaker constant is used for both sulfuric acid and HOMs because it does not noticeably change the model predictions.

Ions and charged clusters collide according to a similar expression as equation (1):where *E* is an enhancement factor to obtain the charged collision kernels (described below). The sticking probability for collisions between a neutral particle and a charged particle, , is:where  is a free parameter and *C* = *C*_B_ or *C*_A_ for biogenic or acid particles, respectively. Ion–ion recombination results in a neutral particle, which may evaporate at small sizes. The model allows partial evaporation of such recombination particles; in this case the cluster divides into monomers and the mass is conserved. The probability of cluster survival after ion–ion recombination, , is:where *C*_+−_ is a free parameter. A power of unity (*S*_+−_ = 1) is used because the data do not constrain this parameter well.

To obtain the charged collision kernels, the neutral–neutral collision kernel is multiplied by size-dependent enhancement factors, *E*:where *K* are the collision kernels and the subscripts refer to the charge of the colliding particles. The charged collision kernels in equation (2) are obtained from ref. 51, which refers to sulfuric acid particles. Because biogenic particles may have different neutral–charged collision kernels, their enhancement factor is left free in the fit:where *f*_0+,0−_ is a free parameter.

Ions, monomers, clusters and larger particles are continually lost by diffusion to the walls and by dilution of the chamber contents with fresh gas mixture. The dilution lifetime is near 3 h (10^−4^ s^−1^), depending on the total sampling rate of all instruments attached to the chamber. The wall loss rate is 1.8 × 10^−3^ s^−1^ for H_2_SO_4_ monomers, and decreases with increasing cluster or molecule diameter as 1/*d*. The same scaling law is used to obtain the wall loss rate for HOMs; that is, it is assumed that HOMs and particles that collide with the walls are irreversibly lost. For experimental runs for which there is a pre-existing population of particles in the chamber at the start of a run due to incomplete cleaning of the chamber, losses to this coagulation sink are accounted for by inserting the initial size distribution into the size bins of the model.

To determine the nucleation rates, the five free parameters of the model (*S*_B_, *S*_A_, *S*_0+,0−_, *f*_0+,0−_ and *C*_+−_) are fitted to the experimental particle concentrations in the PSM1.8 and CPC2.5 versus time. For example, for neutral pure biogenic runs, only one free parameter (*S*_B_) is involved in the fit. The value of *S*_B_ ranges from 12 to 14, *S*_A_ from 4 to 6, *S*_0+,0−_ from 0.1 to 1.0, *f*_0+,0−_ is near 4 and *C*_+−_ is near 10,000 Th. The parameters *C*_B_, *C*_A_, *S*_+−_ and *f*_c_ were determined by a global fit to all runs in the dataset and then subsequently fixed at these values. The fitted threshold masses for *C*_B_ and *C*_A_ are around 1,300 Th and 700 Th, respectively. The parameter *S*_+−_ is set to 1.0 and *f*_c_ is set to 0.52. The time development of the particle number concentrations in both counters throughout all of the nucleation events in our dataset is well reproduced by the model (an example is shown in Extended Data Fig. 4b).

After fitting the data with the model, the nucleation rate *J*_1.7_ is determined as the number of particles that grow to a mobility diameter of 1.7 nm or larger in any time-step, divided by the time increment. In each nucleation run at fixed conditions, the time *t*_max_ is determined at which *J*_1.7_ is maximum; the value of *J*_1.7_ for that run is then calculated as the mean measurement over the interval (*t*_max_ ± 300 s).

There are three major advantages of using a data-driven kinetic model to determine nucleation rates rather than making direct measurements with the PSM1.8 or CPC2.5 data. First, it avoids the need for time derivatives of the data, which are subject to large errors at low counting rates. Second, particle growth rates are determined by kinetics and properly account for growth due to collisions both with monomers and with other particles. The model treatment of the data therefore avoids the exponential sensitivity on experimental growth rates that occurs with other methods^52,53,54,55^. Experimental growth rates are determined from particle counter rise times and have relatively large uncertainties in the 1–3-nm size range. Finally, the model requires consistency between the PSM1.8 and CPC2.5 so the formation rates are experimentally constrained both near the 1.7-nm threshold size and near 3 nm.

### Verification of the model nucleation rates

We performed extensive cross-checks of the nucleation rates obtained with the model by calculating the nucleation rates independently in two additional ways: (1) direct measurements at 1.8 nm using the scanning PSM and (2) CPC2.5 measurements that are stepwise-corrected to 1.7-nm threshold size. Within their experimental uncertainties, the nucleation rates obtained by both these methods agree well with the values obtained with the AEROCLOUD kinetic model.

The stepwise-corrected method is described in detail in ref. 55, but a brief summary is provided here. The nucleation rates are derived from the rate of change of the formation rates, d*N*_CPC_/d*t*, where *N*_CPC_ is the particle number concentration measured with the CPC2.5 above its detection threshold, *d*_th_. The formation rate is corrected in two sequential steps for particle losses to chamber walls, dilution and coagulation: (1) particle losses above *d*_th_ and (2) particle losses during growth from 1.7 nm to *d*_th_. The dilution and wall loss rates are the same as in the kinetic model. To calculate the coagulation rate, the particles are divided into size bins and then the loss rate in each bin *i* is computed by summing the size-dependent collision (coagulation-loss) rate of the particles in bin *i* with those in all other bins. The total coagulation loss rate is then the sum of the particle loss rates in each bin *i*.

Correcting for particle losses during growth from 1.7 nm to *d*_th_ (item (2) above) requires knowledge of the particle growth rate. This is experimentally determined with several instruments, for example, from the appearance times measured in the scanning PSM^56^, which detects particles over a range of threshold diameters between 1 nm and 2.5 nm. The growth rates were also measured over different size ranges with several other instruments, including a fixed-threshold PSM, two DEG-CPCs, a TSI 3776 CPC, an APi-TOF, an NAIS, an nRDMA and a nano-SMPS. The experimental growth rates are parameterized because they cannot be measured sufficiently precisely at each point in time during all events. To determine the nucleation rate at 1.7 nm from the corrected formation rate at *d*_th_, the size interval is divided into *m* log-normally spaced bins, dlog(*D*_p_), chosen to match the spacing of the SMPS bins at larger sizes. The residence time of a particle in each bin is δ*t* = δ*d*_*i*_/(growth rate), where δ*d*_*i*_ is the size of bin *i*. Starting with the measured particle distribution above *d*_th_, the size distribution and formation rate is then extended towards 1.7 nm in a stepwise process. In the first step, using the known loss rates due to the chamber walls, dilution and coagulation, as well as the time δ*t*, the concentration in the largest new bin is calculated, as well as the formation rate into this bin. Using this concentration, the size distribution is updated and the process is repeated until, after *m* steps, the smallest size bin at 1.7 nm is reached, where the nucleation rate is determined.

### The NAIS

The neutral cluster and air ion spectrometer (NAIS)^57^ measures the size distributions of positively and negatively charged particles, and also of total (charged plus neutral) particles, between mobility-equivalent diameters of 0.75 nm and 45 nm. Because the instrument includes two mobility analysers operating in parallel, positive and negative spectra are obtained simultaneously, each with 21 electrometers. Taking into account the internal diffusion losses, the mobility distribution is then calculated in 28 size bins from the measured electrometer currents.

The instrument operates sequentially in three modes: ion, particle and offset mode (one cycle takes 150 s). The aerosol sample first passes through a preconditioning section containing a discharger, an electric filter, a charger and a second electric filter (post-filter). The charger and discharger are corona needles of opposite polarities. In ion mode, the preconditioning unit is switched off and the sample passes through unaffected. In this way, the mobility analysers measure only ions and charged particles from the CLOUD chamber. In particle mode—which was not used for the results reported here—both chargers are switched on and so neutral particles from the CLOUD chamber can be classified. The post-filters improve the measurements by removing residual ions from the charger. In offset mode, the dischargers and corresponding filters are switched on. The sample is charged to the opposite polarity as the subsequent analyser and so no detectable particles can enter. In this way, the noise levels and possible parasitic currents are measured to provide corrections for the preceding ion and particle measurement.

After preconditioning, the aerosol sample is classified in two cylindrical mobility analysers. The central electrode consists of several sections, each at a different fixed electric potential. The particles enter the analysers through a circular slit near the central electrode and are collected at the 21 outer electrodes where they transfer their charge to the connected electrometer and the resulting current is measured. The analysers operate at a sheath flow rate of 60 l min^−1^. Filtered excess air serves as sheath gas to ensure conditions similar to the sample flow. The data inversion that converts the measured electrometer currents to particle concentrations is based on model calculations simulating trajectories of particles with different mobilities, and on calibration measurements of the internal losses. The performance of the NAIS for ion-mobility (size) and concentration measurements is described in refs 58, 59.

### The APi-TOF mass spectrometer

The atmospheric pressure interface time-of-flight (APi-TOF) mass spectrometer^14^ measures the mass-to-charge ratio of positive or negative ions with an inlet at atmospheric pressure. The first stage of the instrument consists of an atmospheric pressure interface (APi) section where ions are focused and guided by two quadrupoles and an ion lens through three chambers at progressively lower pressures down to 10^−4^ mbar. The second stage of the instrument is a time-of-flight (TOF) mass spectrometer at 10^−6^ mbar.

The APi-TOF was connected to the CLOUD chamber via a 1″ (21.7-mm inner diameter) sampling probe shared with the NAIS. A Y-splitter divided the total flow of 20 l min^−1^ equally between the two instruments. The sample flow for the APi-TOF was 0.8 l min^−1^, with the remainder being discarded.

The APi-TOF measurements were made during GCR and *π*^+^ beam runs; that is, the ions were charged by GCRs or charged pions traversing the CLOUD chamber. Because the APi-TOF can measure only one polarity at a time, positive and negative ions were measured in different runs. Different instrument settings were used during the campaigns to optimize detection in the low- or high-mass regions of the spectrum. The data were analysed with *tof Tools*^35^, developed by the University of Helsinki. The tool is implemented in MATLAB and allows complete processing of TOF data: averaging, mass calibration, baseline detection, peak fitting and high-resolution analysis.

### The CI-APi-TOF mass spectrometer

Two nitrate chemical ionization atmospheric pressure interface time-of-flight (CI-APi-TOF) mass spectrometers were used to measure neutral sulfuric acid and HOMs. The instruments were operated by the University of Frankfurt (UFRA-CI) and the University of Helsinki (UHEL-CI); differences between the two instruments are indicated in this section by adding the UHEL-CI characteristics in parentheses after those of the UFRA-CI. The CI-APi-TOF has been described previously^36,37^. The sample air from the CLOUD chamber was drawn in through a 1/2″ stainless steel tube at flow rate of 9 l min^−1^ (10 l min^−1^). An electrostatic filter was installed in front of each instrument to remove ions and charged clusters formed in the chamber. The geometry of both ion sources follows the design of ref. 60, but a corona charger^34^ (X-ray generator) is used for ion generation. Dry air with nitric acid vapour is flushed over the ionizer to generate  ions. The ions are guided into the sample flow with an electric field, where they react with sulfuric acid and HOMs. The reaction time is approximately 50 ms (200 ms) before the ions enter the APi section through a pinhole with a diameter of 350 μm (300 μm). The APi section consists of three consecutive differentially pumped chambers where the pressure is progressively reduced and the ions are focused by two sets of quadrupoles and an ion lens system. The mass-to-charge ratios, *m*/*z*, of the ions that pass through these chambers are measured by a time-of-flight (TOF) mass spectrometer (Tofwerk AG).

The voltage settings in the APi-TOF section influence the mass-dependent transmission efficiency. The transmission curves were determined in a series of calibration measurements in which various perfluorinated acid vapours of different *m*/*z* were passed into the instrument in sufficient amounts to saturate all the primary ions. In this way, a constant ion signal could be generated at each *m*/*z* and so the transmission efficiency could be determined relative to that of the primary ions mass range. The UFRA-CI operated at the same voltage settings for the entire data collection period; the UHEL-CI was operated in a switching mode between two voltage settings optimized for low and high *m*/*z*, respectively.

The raw data were analysed with the MATLAB *tofTools* package^35^. The mass scale is calibrated to an accuracy of better than 10 p.p.m. using a two-parameter fit. The concentration of sulfuric acid is calculated from the ratio of bisulfate ion counting rates (in s^−1^) relative to primary ions as follows:The factor  corrects for losses in the sampling line from the CLOUD chamber. The calibration coefficient, *C*, is determined by connecting the CI-APi-TOF to a well-characterized H_2_SO_4_ generator^61^. The value of *C* depends on the voltage settings in the APi-TOF section and was determined to be 6.5 × 10^9^ cm^−3^ (1.2 × 10^10^ cm^−3^ and 2.8 × 10^9^ cm^−3^ for the high and low *m*/*z* settings, respectively), with an uncertainty of +50%/−33%. The H_2_SO_4_ detection limit is 5 × 10^4^ cm^−3^ or slightly lower.

The concentration of a HOM at *m*/*z* = *i* is calculated as follows:Here,  is the background-subtracted counting rate of the HOM. Background levels were measured by sampling air from the clean CLOUD chamber without any α-pinene present. The factor *T*_*i*_ is the mass-dependent transmission efficiency. The calibration coefficient, *C*, is the same as that obtained for sulfuric acid because HOMs and sulfuric acid were shown to have similar molecular collision rates with the nitrate ions^16^. Furthermore, the binding of  with highly oxidized HOMs is found in the present study to be strong, so clustering should proceed at near the kinetic limit, as it does for  with sulfuric acid. The factor  corrects for losses in the sampling line from the CLOUD chamber. The values were determined for E_1_ and E_2_ separately, using experimentally determined diffusion coefficients, as  and .

The HOM monomers, E_1_, are the background-subtracted sum of the peaks in the *m*/*z* band 235–424 Th; the HOM dimers, E_2_, are the corresponding sum for 425–625 Th. Instrumental contamination peaks are excluded from the band summation, as are peaks assigned to the RO_2_· radical (C_10_H_15_O_6,8,10,12_, which correspond to *m*/*z* = 293 Th, 325 Th, 357 Th and 389 Th). Total HOMs is defined as the sum RO_2_· + E_1_ + E_2_.

### HOM yields

The HOM yields from either ozonolysis or OH· chemistry were calculated by assuming equal production and loss rates during steady-state^16^:where the yield, *γ*_Ox_, is the fraction of α-pinene (AP) oxidation reactions leading to HOM formation, and ‘Ox’ signifies O_3_ or OH·. The values of the rate constants (in cm^3^ per molecule per second) at 278 K for oxidation of α-pinene are  and *k*_AP + OH·_ = 5.84 × 10^−11^, from the International Union of Pure and Applied Chemistry (IUPAC)^62^ (the α-pinene + O_3_ rate constant is updated on the IUPAC website at http://iupac.pole-ether.fr/htdocs/datasheets/pdf/Ox_VOC8_O3_apinene.pdf). The HOM wall loss rate was determined to be 1.1 × 10^−3^ s^−1^, assuming they are irreversibly lost. An additional loss is due to dilution of the chamber contents by makeup gases (0.1 × 10^−3^ s^−1^). The total loss rates for HOMs is then *k*_loss_ = 1.2 × 10^−3^ s^−1^.

During the experiments involving pure OH· chemistry, nitrous acid (HONO) concentrations ranging from 0.5 p.p.b.v. to 3 p.p.b.v. were photolysed by ultraviolet radiation from the fibre optic system to produce OH·. This led to a small contamination of NO in the chamber, which may potentially influence the HOM yield. The OH· concentrations in the CLOUD chamber were estimated using the PTR-TOF measurements of the difference of the α-pinene concentrations with no OH· present (ultraviolet off) and OH· present (ultraviolet on at different intensities). The decrease in α-pinene was due to only OH· reactions, because no O_3_ was present in the chamber during these experiments. The accuracy for [OH·] is estimated to be ±30% (1*σ*) including uncertainties in α-pinene measurements and reaction rate constant, which leads to a systematic scale uncertainty on the HOM production rate, *k*_AP + OH·_[AP][OH·], of ±40% (1*σ*). However, run-to-run uncertainties contribute substantially to the overall uncertainty as indicated by the error bars in Extended Data Fig. 2.

### The SO_2_-CIMS

The SO_2_ chemical ionization mass spectrometer (SO_2_-CIMS) uses  primary ions to convert SO_2_ to , which is then measured in a quadrupole mass spectrometer with an APi interface (Georgia Tech). The general design of the ion source is shown in ref. 60, but the primary ions are generated with a corona discharge^34^. The corona needle holder was modified so that CO_2_, O_2_ and Ar are fed directly over the corona discharge. In this way, direct contact between the N_2_ sheath flow and the discharge needle is avoided, which leads to a reduced contamination by  and maximizes the ratio of  to . The reaction scheme for the ionization of SO_2_ to  can be found in ref. 63. The use of a dry N_2_ buffer flow in front of the pinhole of the mass spectrometer evaporates associated water molecules from  ions, and so sulfur dioxide is detected in the mass spectrum at *m*/*z* = 112 Th ().

The SO_2_ concentration (in p.p.t.v.) is calculated from the ion count rates, *R*_*m*/*z*_, as follows:where *R*_112_ corresponds to the background-corrected ion count rate of  and *R*_60_ is the ion count rate of the primary ion . The calibration factor *C*_S_ was obtained by periodically calibrating the instrument with a SO_2_ gas standard (Carbagas AG) during the campaign. During a calibration, the gas standard was diluted with ultraclean humidified air at 38% relative humidity (the same as that supplied to the CLOUD chamber) to achieve a range of different SO_2_ mixing ratios between 12 p.p.t.v. and 11 p.p.b.v. The calibration factor was found to be 1.3 × 10^5^ p.p.t.v., with an estimated uncertainty of ±11%. The error includes uncertainties in the flow rates during a calibration and in the gas standard concentration, as well as statistical uncertainties. However, we also observed that temperature changes in the experimental hall where the experiments were conducted led to a drift in the  background signal when no SO_2_ was applied to the CIMS. This effect contributes to the overall uncertainty and mainly affects the measurement at low SO_2_ levels (<100 p.p.t.v.), with lower precision in this concentration range. For example, at 30 p.p.t.v. SO_2_, the estimated uncertainty is ±23%, but it becomes progressively smaller with higher SO_2_ levels, reaching ±13% above 100 p.p.t.v. SO_2_. The detection limit of the instrument is 15 p.p.t.v. SO_2_.

### Experimental errors

To determine *J*_1.7_, the measured particle concentrations in the PSM1.8 and CPC2.5 versus time are fitted with the AEROCLOUD model (see above). The nucleation rate error, *σ*_*J*_, has three main components. The dominant error at slow growth rates is due to uncertainties in the PSM1.8 and CPC2.5 detection thresholds for HOM particles^64^. The threshold error components are first determined numerically for each nucleation measurement by performing additional AEROCLOUD fits after shifting the PSM1.8 particle detection threshold by +0.2/−0.1 nm and the CPC2.5 threshold by ±0.4 nm. This provides four fractional *J*_1.7_ errors which are then averaged for each counter to provide a mean fractional uncertainty, *σ*_psm_ and *σ*_cpc_, respectively. The total error due to detection threshold uncertainties, *σ*_thr_, for the combined fit to the PSM1.8 and CPC2.5 data is then:The total fractional *J*_1.7_ error, *σ*_*J*_, is then obtained by adding *σ*_thr_ in quadrature with an experimental error due to run-to-run reproducibility under nominally identical chamber conditions, *σ*_exp_, and an error to account for model approximations, *σ*_model_:where *σ*_exp_ = 30% and *σ*_model_ = 50%.

The concentration of O_3_ is measured with a calibrated instrument and is known to ±10%. The α-pinene concentration in the PTR-TOF is known to ±10%. As discussed above, the uncertainty on SO_2_ is ±13% above 150 p.p.t.v., increasing at lower values to ±23% at 30 p.p.t.v.

For CI-APi-TOF measurements, the run-to-run experimental uncertainties are ±10% for [H_2_SO_4_] and ±20% for [HOM]. However, there is a larger overall systematic error that scales all measurements by the same amount. The systematic scale uncertainty for [H_2_SO_4_] is estimated to be +50%/−33%. This estimate is based on a comparison of [H_2_SO_4_] measurements with a CIMS and a calibrated H_2_SO_4_ generator^61^. The systematic uncertainties for [HOM] have the following sources and fractional errors (1*σ*): sulfuric acid calibration (50%), charging efficiency of HOMs in the ion source (25%), mass dependent transmission efficiency (50%) and sampling line losses (20%). This results in an overall systematic scale uncertainty for [HOM] of +80%/−45%. The uncertainty in the HOM yield from ozonolysis or hydroxyl chemistry is estimated by adding the [HOM] uncertainty in quadrature with the errors for α-pinene (10%), O_3_ (10%), OH· (30%), HOM wall loss rate (6%) and rate constants (35% for the α-pinene O_3_ reaction and 20% for the α-pinene OH· reaction). This results in a mean estimated uncertainty in HOM yield for either ozonolysis or hydroxyl chemistry of +100%/−60%.

### Quantum chemical calculations

To estimate the characteristic binding energies and evaporation rates expected for ELVOC clusters, we chose C_10_H_14_O_7_ (molecular weight of 246) to represent the ELVOC monomer, E_1_, and C_20_H_30_O_14_ (molecular weight of 494) to represent the covalently bound ELVOC dimer, E_2_. Their formation mechanism and structures are shown in Extended Data Figs 3 and 7. To evaluate the effect of charge on the formation of ELVOC clusters, we studied initial molecular clusters of E_1_ and E_2_ that are either neutral or else include an ion of the type , ,  or  (Extended Data Table 1).

We calculated formation Gibbs free energies at 278 K, Δ*G*_278 K_, of different clusters with the MO62X functional^65^ and the 6-31+G(d) basis set^66^ using the Gaussian09 program^67^. The formation Gibbs free energy can be related to evaporation rate as described in refs 68, 69. In previous works^10,15^, we used the method proposed in ref. 68 for calculating the formation free energy of different clusters. However, this method is too computationally demanding for the large clusters of the present study. The MO62X functional has been shown to be well suited to the study of atmospheric clusters^70^. Ref. 70 has shown how reducing the basis set from the largest Pople basis set available (6-311++G(3df,3pd)) to the basis set used in this work (6-31+G(d)) leads to differences in the calculated formation free energies below 1 kcal mol^−1^. Therefore, MO62X/6-31+(d) is a good alternative to the B3RICC2 method^68^ when studying large clusters. We confirmed this by comparing the formation free energies previously calculated^15^ using the B3RICC2 method with those calculated here using the MO62X/6-31+G(d) method. The differences were found to be below 2 kcal mol^−1^.

### Parameterization of the pure biogenic nucleation rate

We parameterized the experimentally measured pure biogenic nucleation rates in a form suitable for global aerosol models. The neutral and ion-induced pure biogenic nucleation rates (in cm^−3^ s^−1^) are parameterized as:where [*n*_±_] = [*n*_+_] = [*n*_−_] is the small-ion concentration of either sign. Expressions for [HOM] and [*n*_±_] are given in equations (7) and (10) below, respectively. The parameters *a*_*n*_ are determined from fits to the data in Fig. 3 and have the values *a*_1_ = 0.04001, *a*_2_ = 1.848, *a*_3_ = 0.001366, *a*_4_ = 1.566 and *a*_5_ = 0.1863, with [HOM] expressed in units of 10^7^ cm^−3^. The parameterized rates are shown by the curves in Fig. 3. The *R*^2^ value of the fit is 0.97. The terms *a*_1–4_ describe simple power laws, whereas the term *a*_5_ accounts for the steepening of the nucleation rate at low HOM concentrations. The nucleation rates are assumed to be independent of temperature, except for the effect of rate constants (equation (6) below), because the experimental measurements exist at only a single temperature.

The HOM concentration in equation (4) is determined from its production and loss rates:where MT represents total monoterpenes. The IUPAC^62^ reaction rate constants (in cm^3^ per molecule per second) for oxidation of α-pinene by ozone and hydroxyl radicals are, respectively:where *T* (in K) is the temperature (the α-pinene+O_3_ rate constant is updated on the IUPAC website at http://iupac.pole-ether.fr/htdocs/datasheets/pdf/Ox_VOC8_O3_apinene.pdf). The HOM yields in each ozone–monoterpene and hydroxyl–monoterpene reaction are  and , respectively. The parameter *k*_HOM_ is the HOM loss rate or, equivalently, the atmospheric condensation sink, CS (in s^−1^). The condensation sink is determined assuming the diffusion characteristics of a typical α-pinene oxidation product (see appendix A1 of ref. 71). Assuming steady-state in equation (5), the HOM concentration becomes:where the HOM yield from ozonolysis is , and from reaction with the hydroxyl radical is  (Extended Data Fig. 2). The HOM yield from ozonolysis is determined from CLOUD measurements in the presence of a hydroxyl scavenger (0.1% H_2_). The HOM yield from reaction with hydroxyl radicals is determined from CLOUD measurements in the absence of ozone, and where photolysed HONO provides the OH· source. Therefore, the experimental measurement of hydroxyl-initiated oxidation is made in the presence of NO_*x*_, as occurs in the atmosphere.

The small-ion concentration in equation (4) is calculated from the steady-state solution of the ion balance equation:where *q* (in cm^−3^ s^−1^) is the ion-pair production rate and *α* is the ion–ion recombination coefficient (in cm^3^ s^−1^). The factor of 2 in equation (4) accounts for nucleation from positive and negative ions. For the CLOUD GCR data, *q* = 1.7 cm^−3^ s^−1^. Terrestrial radioactivity such as radon contributes additional ionization in the boundary layer over land masses^72^. The ion loss rate, *k*_i_, is due to the condensation sink, CS, and ion-induced nucleation:where *J*_iin_/(2[*n*_±_]) is given by equation (4) and the steady-state concentration of small ions is, from equation (8):From equations (8) and (9), *J*_iin_ saturates at 2*q* at high nucleation rates (see Fig. 3).

## Figures and Tables

**Extended Data Table 1:**
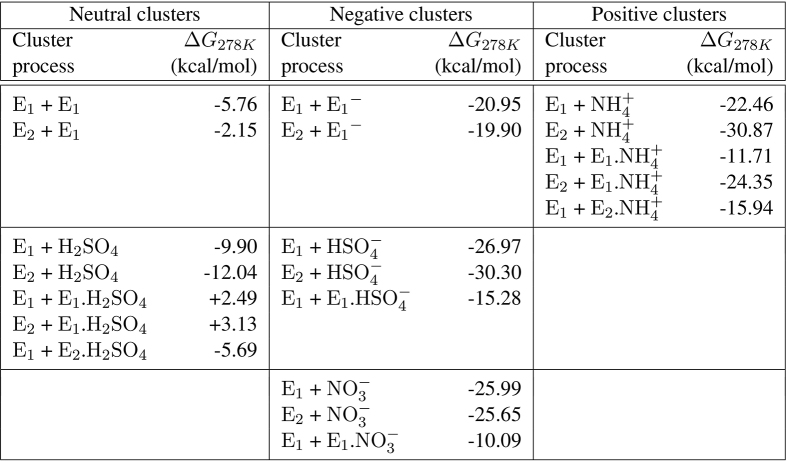
Quantum chemical calculations of ELVOC cluster formation energies

Formation Gibbs free energies at 278 K, Δ*G*_278 K_, for neutral, negatively charged and positively charged ELVOC clusters. The cluster processes indicate the incident E_1_/E_2_ vapour molecule + the target cluster. Quantum chemical calculations made at other temperatures (not shown) indicate that the binding energies strengthen by −1.0 kcal mol^−1^ per 20 K reduction in temperature. The uncertainty in the calculated energies is less than 2 kcal mol^−1^. Our calculations indicate the following approximate order for different functional groups to contribute to the cluster binding energies involving  or H_2_SO_4_ (starting with the strongest): (i) carboxylic acids, R–C(=O)–OH; (ii) hydroxyls, R–OH; (iii) hydroperoxy acids, R–C(=O)–O–OH; (iv) hydroperoxides, R–O–OH; and (v) carbonyls, R–(R′–)C=O. In the case of , the main interacting group is carbonyl, independently of which other groups are attached to it; therefore ammonium will form stronger clusters with carboxylic acids, hydroperoxy acids or carbonyls than it will with hydroxyls or hydroperoxides.

## Related audio

Davide Castelvecchi talks to the researchers making clouds in the lab.

## PowerPoint slides

PowerPoint slide for Fig. 1
PowerPoint slide for Fig. 2
PowerPoint slide for Fig. 3
PowerPoint slide for Fig. 4
