# Supplementary material for: Ion-induced nucleation of pure biogenic particles
Source: Nature. 2016 May 25;533(7604):521–6. doi: 10.1038/nature17953 (PMC8384037; doi:10.1038/nature17953)
Supplement: Supplementary file 5 — PowerPoint slide for Fig. 4 [file 41586_2016_BFnature17953_MOESM53_ESM.ppt]

## Slide 1
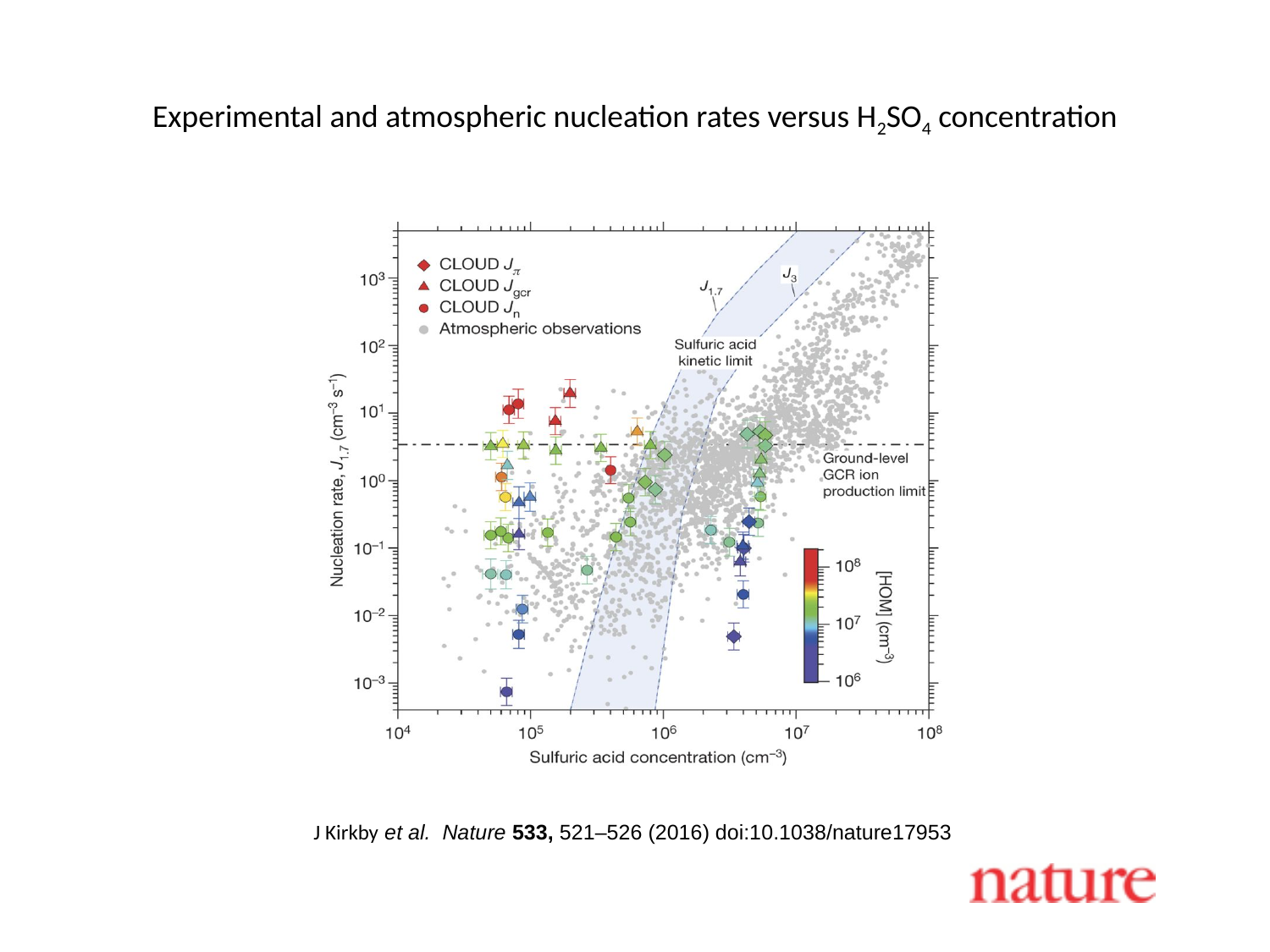

# Experimental and atmospheric nucleation rates versus H2SO4 concentration
J Kirkby et al. Nature 533, 521–526 (2016) doi:10.1038/nature17953
